# Coronary artery bypass grafting in patients with malignancy: a single-institute case series of eight patients

**DOI:** 10.1186/s12893-022-01805-7

**Published:** 2022-10-13

**Authors:** Ming-Kui Zhang, Han-Wen Zhang, Qing-Yu Wu, Hui Xue, Li-Xin Fan

**Affiliations:** Heart Center, First Hospital of Tsinghua University, No.6, 1st street, Jiuxianqiao, Chaoyang District, 100016 Beijing, China

**Keywords:** Coronary artery disease, Oncology, Surgery, Coronary artery bypass grafting

## Abstract

**Background:**

The surgical strategy among patients with malignancy and coronary artery disease (CAD) remains controversial. In this study, we present the experiences of coronary artery bypass grafting (CABG) in patients with malignancy and analyzed the treatment outcomes.

**Methods:**

From January 2011 to October 2021, eight patients combined with coronary artery disease and malignancy, six of them with three-vessel disease and two with anterior descending branch lesions on coronary angiography. The age ranged from 54 to 73 years (61.8 ± 7.7years). Four patients underwent CABG and staging for surgical oncology, and 2 patients underwent CABG and surgical oncology simultaneously. Four patients underwent CABG procedure with cardiopulmonary bypass (on-pump CABG), and the other patients underwent the procedure without cardiopulmonary bypass (off-pump CABG). All patients were followed up for 3 to 96 months (40.4 ± 31.5 months) postoperatively.

**Results:**

The mean number of grafts was 2.6 ± 1.1, there was no in-hospital death, postoperative myocardial infarction, and stroke. Among the eight patients, one patient received chemotherapy and radiation before bypass surgery, which occurred postoperatively pulmonary infection, and the rest of 7 patients had no major adverse cardiovascular events during follow-up periods.

**Conclusion:**

Based on the results of the present study, simultaneous or staged CABG and oncologic surgery according to the TNM stage of the tumor and cardiac assessment is an effective treatment for patients with severe CAD combined with malignancy.

## Background

Cardiovascular disease and malignant disease are the leading causes of death in many parts of the world. Both diseases have similar populations and face the same risk factors (e.g. use of tobacco, aging, and obesity) so the simultaneous presence of both diseases is not rare . The prevalence of malignancy and coronary heart disease among male patients was about 7% [1–3]. The presence of malignancy in cardiovascular series ranged between 1.9 and 4.2%, and cardiovascular disease was present in 25% of malignant patients [4–7]. Meanwhile, radio- and chemo-therapy in malignancy also impacted the heart [8]. Cardiac surgery for patients with malignancy is also a major challenge for cardiac surgeons, and the reduced predictable survival time for malignancy has an impact on their surgical choices. Studies have shown that myocardial revascularization in patients with combined coronary artery disease can make subsequent oncological surgery or chemotherapy safer and increase the patient’s long-term survival [9, 10].

The effect of cardiopulmonary bypass (CPB) on malignant cell growth and dissemination is not clear [11, 12]. Some studies demonstrated that the use of CPB could inhibit both cell-mediated and humoral immunity [13]. For these reasons, there are still many conflicting treatment strategies for patients with coronary artery disease and malignancy.

The aim of this study was to analyze the outcome of surgical treatment of severe coronary artery disease in combination with malignancy, to identify its risk factors and their impact on immediate and long-term outcomes, and to further explore its surgical treatment strategies.

## Methods

### Patients

This retrospective study was approved by the Ethics Committee of First Hospital of Tsinghua University (No.20,220,008). We reviewed the hospital’s database and analyzed the chart datum, surgical records, and imaging data. From January 2011 to November 2021, a total of 8 cases, including 6 males and 2 females, aged 54–72 (61.8 ± 7.7) years, with malignancy combined with severe coronary artery disease, were treated at the Heart Centre of the First Hospital of Tsinghua University. Malignant tumors include lung, rectum, colon, bladder, esophagus, thymoma, gastric lymphoma, and parotid lymphoma (parotid non-Hodgkin’s lymphoma surgery and tissue diagnosis in another hospital). Three patients had the symptoms of myocardial ischemia, one patient had a history of myocardial infarction. The remaining 4 patients had no symptoms of myocardial ischemia before malignancy was diagnosed. On coronary angiography, six patients had the three-vessel disease, including two cases of combined left main coronary artery disease. Two cases had left anterior descending artery lesions. Four patients had ST-segment downward shift and T-wave hypoplasia or inversion on electrocardiogram (ECG) at presentation, and one case had pathological Q waves. There were two cases with New York Heart Association cardiac function class I, 5 cases with class II, and one case with class III. The echocardiographic examination of the left ventricular end-diastolic diameter was 44–52 (45.6 ± 4.3) mm, and the left ventricular ejection fraction was 31–65% (54.1 ± 10.5%). Preoperative clinical characteristics are shown in Table 1. All patients underwent a routine clinical examination before surgery.

**Table 1:** Preoperative characteristics of the patients

Variable	Number (n = 8)
Age (years)	61.8 ± 7.7
Gender (M:F)	6:2
Left ventricular ejection fraction (%)	54.1 ± 10.5
Unstable angina	4
Atrial fibrillation	0
Diabetes	3
Hypertension	5
Peripheral arterial disease	0
Hyperlipidemia	7
Chronic obstructive pulmonary disease	0
Smoking	4

M = Male; F = Female; CAD = coronary artery disease

## Surgical approach

Indications for CABG: (i) according to the preoperative tumor, node, and metastasis (TNM) stage, if the tumor was in an early stage (stage I or II) or in patients who were amenable to radical surgery, and the estimated prognosis is > 1 year, and/or (ii) when CAD precluded oncologic treatment (surgery or chemotherapy) and that was mandatory to improve the patient’s prognosis [10]. Exclusion criteria: tumor metastasis; other concomitant cardiovascular diseases; important organ failure; and other contraindications to surgery. The CABG procedure was performed using on-pump or off-pump as determined by the patient’s condition and the surgeon’s experience.

Conventional coronary artery bypass grafting (CABG) was conducted via a standard median sternotomy and CPB was established between the aorta and right atrium. Cardioplegia was given in an antegrade manner. Off-pump CABG procedure was performed via either small anterolateral thoracotomy for revascularization of the left anterior descending artery (LAD) using the left internal mammary artery (LIMA), or median sternotomy for multiple revascularizations. The LIMA was used as a bypass graft for the left anterior descending branch in all patients, and the saphenous veins as bypass grafts for the other target vessels. The off-pump CABG procedure was performed using a coronary target vessel local stabilization device (Medtronic Inc., Minneapolis, MN, USA) for compression and fixation, and the proximal coronary artery was blocked with a silicone band. A 7 − 0 polypropylene suture was used for the distal coronary artery anastomosis and a 6 − 0 suture for the proximal end.

## Postoperative treatment and follow-up

After coronary artery bypass grafting surgery, all patients received appropriate anti-tumor therapy and were analyzed for cardiac adverse events. The current status of the patients was recorded using outpatient visit notes or telephone follow-up.

## Results

Four patients underwent conventional CABG and 4 patients underwent off-pump CABG. Four patients underwent the staged technique, and the lapse time between cardiac operations and cancer operations varied from 1 to 3 months. One patient with malignant gastric lymphoma and one patient with parotid non-Hodgkin’s lymphoma received standardized chemotherapy before cardiac operations. The tumor location and histological diagnosis are described in Table 2. One patient with a postoperative sternal fracture was healed after the re-fixation of the sternum. There were no mediastinal infections and no in-hospital deaths, and all patients were discharged from the hospital. The perioperative and postoperative conditions are shown in Table 3.

**Table 2:** Patient profiles

Case	Age Sex	Coronary lesion	Location of tumor	CABG type	Interval days	Anti-tumor therapy	Tissue diagnosis	Stage	Prognosis
1	55 M	1VD	Colon	CABG×1 Off-pump	One-stage	Right hemicolectomy	Adenocarcinoma	T_is_N_0_M_0_ Stage 0	Alive
2	58 M	3VD	Rectum	CABG×3 Off-pump	90	Chemotherapy Rectal resection	Adenocarcinoma	T_3_N_2_M_0_ Stage III A	Alive
3	73 M	3VD	Stomach	CABG×3 Off-pump	N/A	Chemotherapy	Lymphoma	T_1_N_0_M_0_Stage I	Alive
4	57 M	3VD	Parotid	CABG×4	N/A	Chemotherapy Radiation	Lymphoma	Uncertain	Death
5	54 M	3VD	Bladder	CABG×3	26	Chemotherapy Bladder resection	Urothelial carcinomas	T_1_N_0_M_0_ Stage I	Alive
6	72 F	3VD	Right Lung	CABG×3	89	Right upper lobectomy	Adenocarcinoma	T_2_N_0_M_0_ Stage I	Alive
7	67 F	3VD	Esophagus	CABG×3	30	Esophagectomy	Squamous cell carcinoma	T_2_N_0_M_0_ Stage Ib	Alive
8	58 M	1VD	Thymus	CABG×1 Off-pump	One-stage	Thymectomy Lung wedge resection	Malignant thymoma	T_3_N_0_M_0_ Stage III	Alive

CABG = coronary artery bypass grafting; VD = vessel disease;

**Table 3:** Intraoperative and postoperative variables

Variable	Number (n = 8)
No. of distal anastomosis	2.6 ± 1.1
LIMA	8
Drainage (ml/24 h)	505.1 ± 194.2
Re-exploration	1
Blood transfusion (units/patient)	1.6 ± 1.2
Mechanical ventilation > 8 h (%)	37.5
Length of ICU stay > 48 h (%)	25.0
Length of hospital stay (days)	13.9 ± 9.2
Perioperative MI	0
Postoperative stroke	0
Postoperative ARDS	1

LIMA = left internal mammary artery; ICU = intensive care unit; MI = myocardial infarction; ARDS = acute respiratory distress syndrome

One patient with malignant non-Hodgkin’s lymphoma who received radiation therapy and also had pulmonary interstitial fibrosis and poor respiratory function had several postoperative pulmonary infections and later died of pulmonary infection. Seven patients recovered well without tumor recurrence or metastasis, and no cardiovascular adverse events such as angina pectoris, acute myocardial infarction, ventricular arrhythmias, sudden cardiac death, or heart failure occurred during follow-up periods.

## Discussion

With an aging population and improved diagnostic techniques, a significant increase in patients with malignancy combined with coronary artery disease has been found, but perioperative cardiac complications are a major cause of death in patients undergoing non-cardiac surgery for severe coronary artery disease, the treatment strategy for patients with malignancy combined with coronary artery disease remains a challenge for cardiac surgeons [14]. Firstly, for patients with tumors treated with cytotoxic chemotherapy before cardiac surgery, this can easily cause cardiotoxicity and result in cardiac injury. The studies have found that chemotherapy drug-related cardiotoxicity is one of the main causes of death in patients, especially with anthracyclines (e.g. adriamycin) being the most common [2]. In the present study, one patient with gastric lymphoma underwent coronary artery bypass grafting after 3 months of chemotherapy, the preoperative assessment revealed a significant decrease in cardiac function compared to pre-chemotherapy, with an ejection fraction of less than 30%. In addition, radiation therapy is prone to complications of pericarditis, myocardial injury, valve injury, lung injury, and adverse cardiac events, especially for patients with ischemic heart disease [15–17]. One patient in our study with non-Hodgkin’s lymphoma with chemo- and radiotherapy-related pulmonary interstitial disease, recurrent respiratory insufficiency, and pulmonary infections after coronary artery bypass surgery. For oncology patients with combined unstable angina and acute myocardial infarction, they can receive coronary stenting before oncology surgery, but all patients require antiplatelet therapy after stent implantation and complete re-endothelialization after stenting takes approximately 4 weeks to 1 year, and antiplatelet drugs must meet the needs of the regimen or they are prone to in-stent thrombosis [18]. Studies have shown that patients who undergo non-cardiac surgery early after stenting have a significantly higher mortality rate due to in-stent thrombosis [19, 20].

In critical patients with left main coronary artery or three-vessel disease, coronary artery bypass surgery has significant advantages in reducing the incidence of adverse cardiac events during oncologic surgery and preventing thrombosis. Simultaneous coronary artery bypass operation and tumor resection have the advantages of economic benefits, less reoperation trauma, and no delay in tumor surgery, but also have the disadvantages of prolonged operative time and easy co-infection. One study found that simultaneous CABG and lung tumor resection is a safe and effective method, while also allowing earlier completion of lung surgery and avoiding complications caused by delayed surgery [21]. Zhang and colleagues [22] found that simultaneous surgery for CABG and esophageal cancer is safe and reliable. In our study, two cases of simultaneous CABG and tumor surgery, including one case each of colon cancer and malignant thymoma, were performed without complications related to perioperative incisional infection, severe bleeding, or a lung infection. Patients with tumors undergoing staged CABG and tumor surgery had the advantage of a low incidence of adverse cardiovascular events, the interval between the two procedures ranged from 5 to 60 days, and it was also considered that there was no significant correlation between the recurrence or metastasis of the tumor and no correlation to the time between staged procedures [23]. In this study, four patients underwent coronary artery bypass surgery (esophageal cancer, bladder cancer, lung cancer, and rectal cancer) and staged radical tumor surgery, with an interval of approximately 1–3 months between the two procedures, and there were no in-hospital deaths, and no tumor metastasis or recurrence was found during the follow-up periods, and no cardiovascular adverse events such as angina pectoris, acute myocardial infarction, ventricular arrhythmia, sudden cardiac death or heart failure occurred.

The effect of extracorporeal circulation on the growth and spread of malignant tumor cells is not fully understood. It has been suggested that extracorporeal circulation directly suppresses humoral and cellular immunity in patients, thereby affecting the spread of tumor cells. Tønnesen’s study demonstrated that extracorporeal circulation inhibits the cytotoxic function of natural killer (NK) cells against tumor cells and causes a decrease in complement, lymphocytes, and neutrophils [24]. Chen and colleagues [25] found that extracorporeal circulation was acceptable for the risk of distant metastases after complete resection of thoracic malignancies in 14 cases of massive thoracic tumors resected under extracorporeal circulation with long-term follow-up. In addition, several reports comparing on-pump and off-pump CABG in cancer patients have shown no significant major adverse effect of extracorporeal circulation on cancer recurrence and late-stage survival [10, 11, 26].

In our study, four patients underwent myocardial revascularization under extracorporeal circulation and four patients without extracorporeal circulation. All patients had no postoperative distant metastases. This study demonstrated that complete myocardial revascularization under extracorporeal circulation can reduce the incidence of cardiac adverse events in the perioperative period and surgical oncology. However, the absence of distant metastases may also be related to the early TNM stage of the tumor in this study.

There were several limitations to the study. First, this is a retrospective study with a small sample number. Recall bias in the clinical course cannot be denied. More cases of the malignant disease combined with CAD are needed to confirm clinical data. Secondly, the distribution of diseases in this study is scattered, including gastrointestinal, respiratory, urinary, and thymic malignancies, and malignant lymphoma and further classification studies are a challenge. The value of TNM staging of tumors in the selection of treatment strategies for patients with malignancy combined with coronary artery disease requires further research.

## Conclusion

Coronary artery bypass surgery can reduce the incidence of cardiovascular adverse events in patients with malignancy on antineoplastic therapy and its perioperative period. Depending on the patient’s condition, simultaneous coronary artery bypass grafting surgery and surgical oncology treatment is a safe and effective treatment option.

## Data Availability

The datasets used and/or analyzed during the current study are available from the corresponding author on reasonable request.

## Abbreviations

CAD: Coronary artery disease.
CABG: Coronary artery bypass grafting.
CPB: Cardiopulmonary bypass.
ECG: Electrocardiogram.
LAD: Left anterior descending artery.
LM: Left main coronary artery disease.
VD: Vessel disease.
LIMA: left internal mammary artery.
ARDS: Acute respiratory distress syndrome.
ICU: Intensive care unit.
MI: Myocardial infarction.
